# Identification of novel PHGDH inhibitors based on computational investigation: an all-in-one combination strategy to develop potential anti-cancer candidates

**DOI:** 10.3389/fphar.2024.1405350

**Published:** 2024-08-27

**Authors:** Yujing Xu, Zhe Yang, Jinrong Yang, Chunchun Gan, Nan Qin, Xiaopeng Wei

**Affiliations:** ^1^ Tianjin Key Laboratory on Technologies Enabling Development of Clinical Therapeutics and Diagnostics, School of Pharmacy, Tianjin Medical University, Tianjin, China; ^2^ Tianjin Mental Health Center, Department of Pharmacy, Tianjin Anding Hospital, Tianjin, China; ^3^ School of Medicine, Quzhou College of Technology, Quzhou, China

**Keywords:** PHGDH, cancer, 3D-QSAR pharmacophore model, ADME/T, molecule docking, molecular dynamics simulation

## Abstract

**Objective:**

Biological studies have elucidated that phosphoglycerate dehydrogenase (PHGDH) is the rate-limiting enzyme in the serine synthesis pathway in humans that is abnormally expressed in numerous cancers. Inhibition of the PHGDH activity is thought to be an attractive approach for novel anti-cancer therapy. The development of structurally diverse novel PHGDH inhibitors with high efficiency and low toxicity is a promising drug discovery strategy.

**Methods:**

A ligand-based 3D-QSAR pharmacophore model was developed using the HypoGen algorithm methodology of Discovery Studio. The selected pharmacophore model was further validated by test set validation, cost analysis, and Fischer randomization validation and was then used as a 3D query to screen compound libraries with various chemical scaffolds. The estimated activity, drug-likeness, molecular docking, growing scaffold, and molecular dynamics simulation processes were applied in combination to reduce the number of virtual hits.

**Results:**

The potential candidates against PHGDH were screened based on estimated activity, docking scores, predictive absorption, distribution, metabolism, excretion, and toxicity (ADME/T) properties, and molecular dynamics simulation.

**Conclusion:**

Finally, an all-in-one combination was employed successfully to design and develop three potential anti-cancer candidates.

## 1 Introduction

**FIGURE 1:**
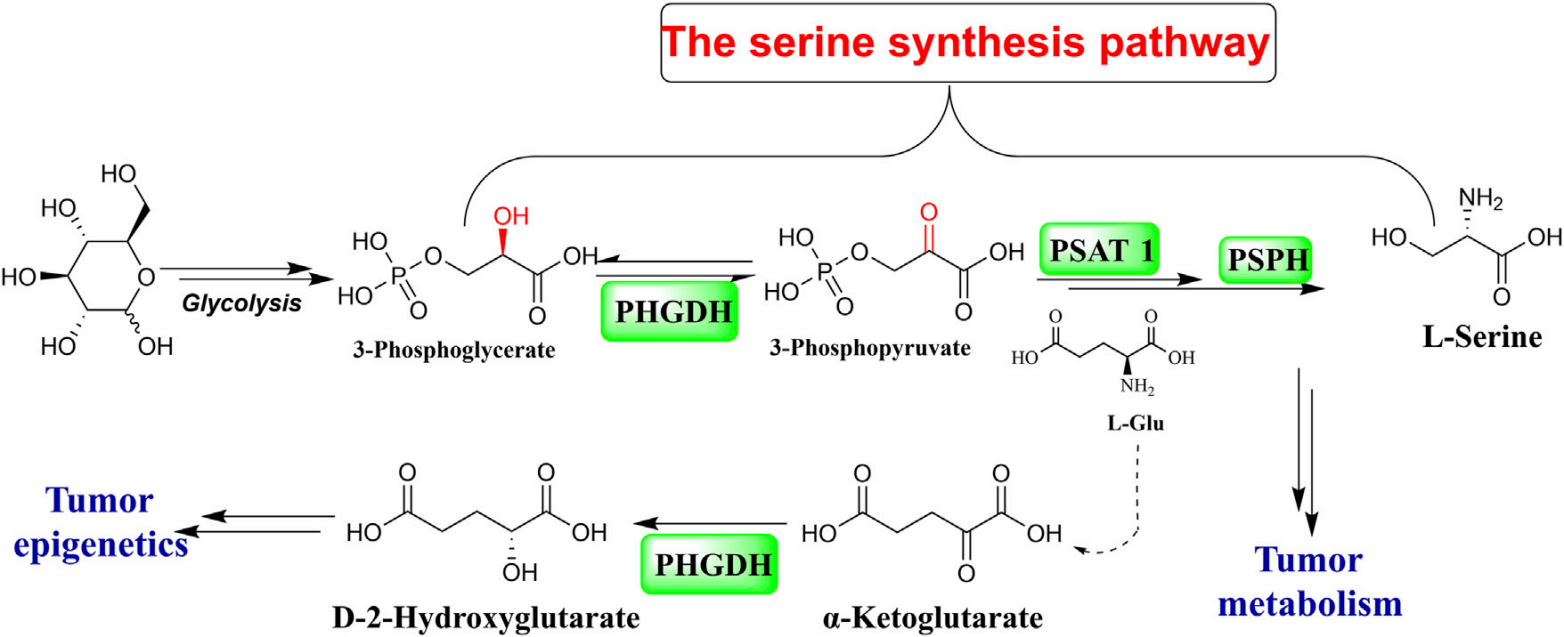
The serine synthesis pathway.

As the primary source of a single carbon unit and the third-largest metabolite, serine is taken up by cancer cells to fuel their unregulated and rapid proliferation. The glycolytic intermediate 3-phosphoglycerate (3-PG), as the substrate for three consecutive enzymatic reactions, makes up an intracellular material for *de novo* serine production. Phosphoglycerate dehydrogenase (PHGDH) catalyzes the first stage of the reactions that convert 3-PG to 3-phosphohydroxypyruvate (3-PPyr) (Figure 1) (El-Hattab, 2016). PHGDH is abnormally expressed in various malignant tumor cells, including hepatocellular carcinoma, breast cancer, melanoma, lung cancer, glioma, colon carcinoma, Ewing sarcoma, pancreatic cancer, leukemia, thyroid carcinoma, multiple myeloma, lymphoma, and gastric and bladder cancer (Zhao et al., 2021). PHGDH dysregulation is a prevalent feature in a significant portion of malignancies with respect to their generation, proliferation, differentiation, and metastatic progress, which suggests that targeting PHGDH is a very promising direction in anti-cancer drug discovery (Rohde et al., 2018; Zhang et al., 2022; Gao et al., 2023).

**FIGURE 2:**
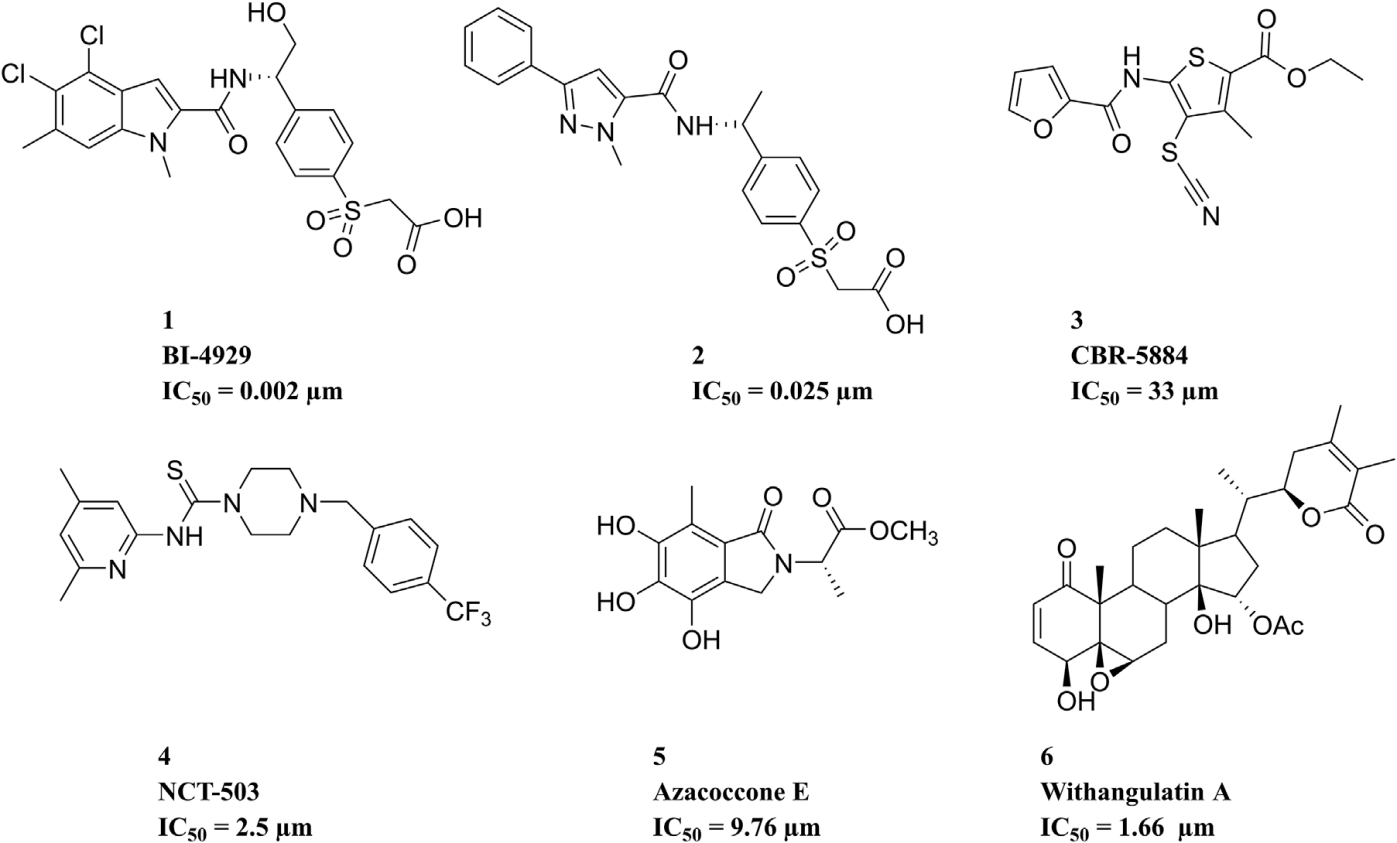
Structures of representative potential PHGDH inhibitors that have been reported.

Over the past decade, numerous potential inhibitors have been reported (Figure 2). Weinstabl et al. (2019) reported a highly potent and selective PHGDH inhibitor, **BI-4924**, which achieved ∼100 μM hits and was optimized to single-digit nanomolar potency. Prior to this, a series of pyrazole-5-carboxamides analogs representing compound **2** were identified (Zhao et al., 2021). **CBR-5884**, reported by Mullarky et al. (2016), contained functional groups that targeted the sulfhydryl moiety and could decrease *de novo* serine synthesis by reacting with the PHGDH cysteine residue. **NCT-503** selectively affected the PHGDH oligomerization state in PHGDH-dependent cell lines and xenograft tumors (Rohde et al., 2018). Natural PHGDH inhibitors, such as **Azacoccone E** and **Withangulatin A**, were isolated from food and microorganisms (Guo et al., 2019; Zheng et al., 2019). Even though these emerging PHGDH inhibitors have not yet been employed in clinical trials, the rapid discovery of structurally novel potential PHGDH inhibitors with safety profiles is particularly urgent. Therefore, 3D-QSAR-pharmacophore-based virtual screening was performed in this study to identify novel PHGDH inhibitors.

## 2 Materials and methods

### 2.1 3D-QSAR pharmacophore model generation

We performed a literature search and collected 31 compounds with corresponding IC_50_ values ranging from 0.002 µM to 68 µM (Supplementary Figures S1, S2) (Spillier and Frederick, 2021; Zhao et al., 2021; Zhang et al., 2022; Gao et al., 2023). The Generate Training and Test Data of Discovery Studio (version 4.0, Biovea Inc., Omaha, NE, United States) protocol offered a random way to split a data set into 22 training set compounds and nine test set compounds by setting the training set percentage to 80. The training set was used to create a 3D-QSAR pharmacophore model, and the test set was reserved to assess the quality of the model. The Feature Mapping protocol computed all possible pharmacophore features, including hydrophobic (HYB), hydrogen bond donor (HBD), hydrogen bond acceptor (HBA), negative ionizable (NI), and ring aromatic (RA). The uncertainty value of all compounds was fixed at 1.5, and IC_50_ values of individual compounds were selected as an active property before hypothesis generation. Test set and Fisher validations with a confidence level of 95% were applied to validate the models. Other parameters were kept as default. The 3D-QSAR pharmacophore model was generated by running the 3D-QSAR Pharmacophore Generation protocol of Discovery Studio, which requires collecting chemically diverse training set compounds with the same bioassay value (Guner et al., 2004). The activities of the input training set (0.003–68 µM) and test set (0.002–39.6 µM) spanned five orders of magnitude. The best model among the 10 generated pharmacophores was selected by combining parameter correlation coefficient (R), root mean square deviation (RMSD), null cost, total cost, error, and fit values (Zhang et al., 2021).

### 2.2 Pharmacophore validation

The three techniques for validating the pharmacophore models were cost analysis, test set analysis, and Fischer randomization tests. Three types of costs are reported: total cost, null cost, and fixed cost in the HypoGen algorithm (Li et al., 2000). Generally, ∆Cost (Null cost − Total cost) is important in assessing the pharmacophore model. A ∆Cost of more than 60 bits suggests a significant correlation. A ∆Cost in the range of 40–60 bits means the model falls within the prediction range of 70%–90%. Predicting correlation likelihood will be difficult if the cost difference is less than 40 bits (John et al., 2011). The Fischer randomization method is essential to establish a structure–activity relationship between the structures and biological activity of the training set. The hypotheses were validated using the Fischer randomization approach, which rearranged the activity values of the training set molecules to yield 19 random spreadsheets with 95% confidence levels (Zhang et al., 2021). The pharmacophore model was also validated by inputting the test set consisting of nine compounds. All test and training set compounds were constructed and minimized using comparable procedures.

### 2.3 High-throughput virtual screening

The virtual screening compounds were downloaded from the Life Chemicals HTS (https://lifechemicals.com/), which consist of 3.04 million structurally novel molecules designed on the basis of promising drug-like scaffolds, carefully selected building blocks, and advanced cheminformatics approach, 460,160 compounds from the Enamine Hit Locator Library (https://enamine.net/compound-libraries/diversity-libraries), and 47,490 compounds from the ChemDiv 3D-pharmacophore database (https://www.chemdiv.com/catalog/screening-libraries/). The virtual screening based on 3D-QSAR pharmacophore was carried out in the Ligand Pharmacophore Mapping module, which compares a set of ligands to a selected pharmacophore. The relevant ligand-3D-QSAR pharmacophore mappings were exported and aligned to the pharmacophore.

### 2.4 ADMET property prediction

Absorption, distribution, metabolism, excretion, and toxicity (ADMET) properties were predicted on AdmetSAR2 (http://lmmd.ecust.edu.cn/admetsar2/) (Yang et al., 2019) and Swiss-ADME online web (https://www.swissadme.ch) (Daina et al., 2017). The toxicities were predicted in ProTox-II (https://tox-new.charite.de/protox_II/), a virtual lab for predicting the toxicities of small compounds (Banerjee et al., 2018).

### 2.5 Molecular docking

Molecular docking was performed by using LibDock in Discovery Studio and AutoDock in AMDock software (Version 1.6.1) based on two different algorithms and scoring functions. The LibDock program developed by Diller and Merz (2001) is based on a binding site comprising lists of polar and apolar hot spots and presents docking results as a LibDock score (Raychaudhury et al., 2023). The protein was prepared by removing atomic clashes, water molecules, and unnecessary atoms, deleting alternate conformations, inserting the missing atoms in incomplete residues, and adding hydrogen. The site sphere was defined from PDB site records by Discovery Studio software. The Prepare Ligands module of Discovery Studio was utilized to generate the three-dimensional structures of anticipated bioactive small molecules. AMDock software (Version 1.6.1), which integrates with Autodock Vina, AutoDock4, and AutoDock4Zn, in which Autodock was based on an empirical free-energy force field and rapid Lamarckian genetic algorithm (Fuhrmann et al., 2010) search method, was used to docking study. The exported binding energy was used to assess the receptor–ligand affinity (Forli et al., 2016). Its graphical tool is simple to use and assists in molecular docking studies. The ligands and proteins were prepared by running the Prepare Input module. The co-crystal structure of PHGDH in complex with BI-4924 (PDB ID: 6RJ6) (Weinstabl et al., 2019) was downloaded from the PDB database (https://www.pdbus.org/). The search space was defined from the Center on Hetero: a box (Center: 18.66, −10.27, −1.74; Size: 24, 24, 24) was placed on the geometric center of an existing ligand. Subsequently, the docking simulations were run using Autodock (Valdes-Tresanco et al., 2020).

### 2.6 Molecular dynamics (MD) simulation

The binding stability and intermolecular interactions of the target macromolecule with promising hits after the docking process were assessed using Desmond2023-1 (Schrodinger, LLC, New York, NY, 2024) with OPLS_2005 force field tools (Kaminski et al., 2001) from a dynamic point of view. The orthorhombic box with the TIP3P water model (Mark and Nilsson, 2001) was used to predefine a 10 Å × 10 Å × 10 Å buffer region between the complex and box sides. The solvated system was neutralized with Na^+^ and Cl^−^ ions. The system builder panel allows minimization, which in turn allows the system to relax into a local energy minimum. Subsequently, elapsed 100-ns MD simulations were carried out at a periodic boundary condition, and the recording interval was set to 100 ps, while the ensemble class was set to NPT (T = 300 K, 1 atm pressure). Finally, approximately 1,000 frames were obtained, and the stability of ligand–receptor complexes was assessed by calculating several important parameters such as RMSD and root mean square fluctuations (RMSF). Their interactions also were analyzed when the simulation jobs were completed (Lanka et al., 2023).

### 2.7 Growth scaffold

**Hit1**, with the lowest estimated IC_50_ value (0.0016 µM), was selected as the most promising lead compound. We performed reaction-based ligand enumeration within the PHGDH active pocket for lead optimization by using the Grow Scaffold protocol of Discovery Studio. Based on isosterism of the sulfonic acid moiety, the carboxyl was used to replace the sulfonic acid moiety in **hit1** based on rational synthesizability. The intermediate could be formed by Claisen–Schmidt condensation using 2,5-dioxopyrrolidin-1-yl succinic acid as the starting material. Beginning with the intermediate position in the binding site of the receptor, the hydroxyl was selected to act as a reaction vector for the enumeration. Then, the amide synthesis and esterification reactions were selected. Other parameters were kept as default.

## 3 Results and discussion

### 3.1 Pharmacophore model generations

3D-QSAR pharmacophore models were generated from the features of known compounds that correspond to their activity, which could elucidate the spatial arrangement of chemical features of active compounds and facilitate the quick and effective discovery of promising hit compounds through ligand-based virtual screening (Yu et al., 2015; Desai et al., 2023).

**FIGURE 3:**
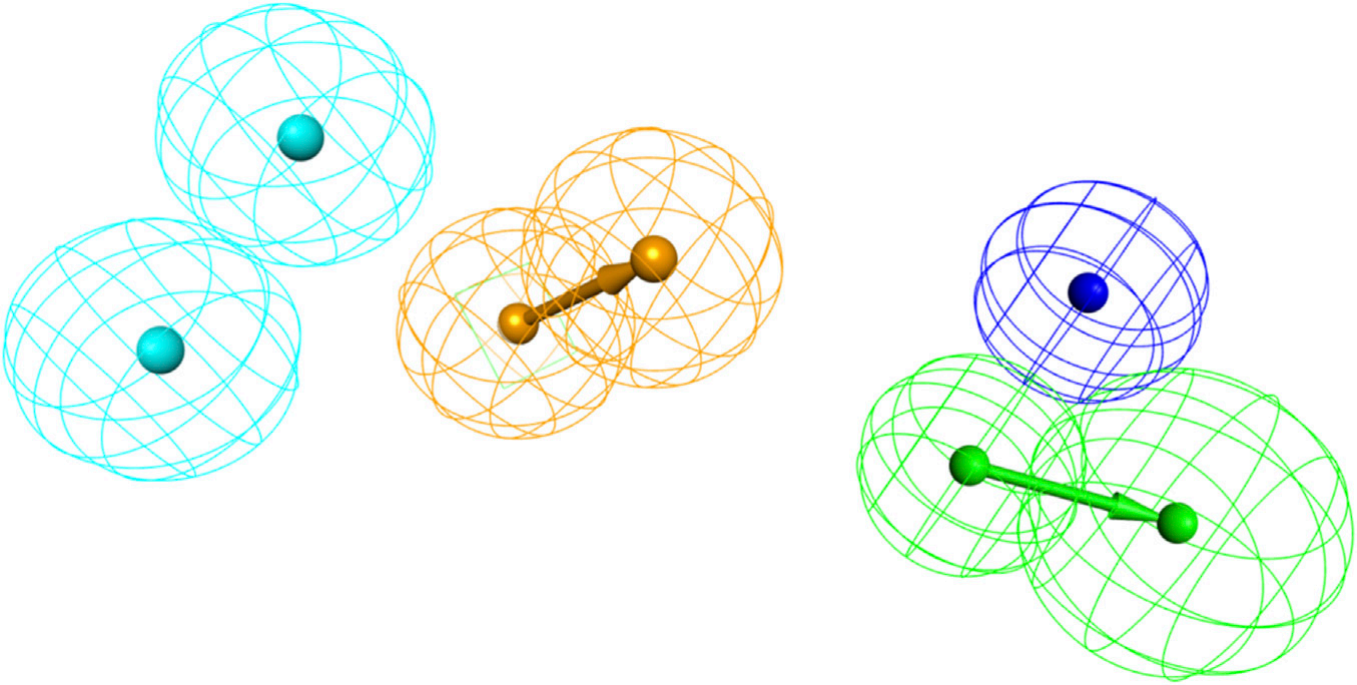
The best pharmacophore model (Hypo_2); hydrogen bond acceptor (HBA, in green); hydrophobic feature (HYD, in blue); ring aromatic (RA, in brown); negative ionizable (NI, in dark blue).

The statistical parameters and chemical features of ten pharmacophore models are shown in Table 1. The total cost of the generated pharmacophore models ranged from 133.247 to 203.149, with a null cost of 564.644 and a fixed cost of 74.9106. Typically, the selected hypothesis has a significant total cost value, a low RMSD, a high correlation coefficient, and the highest cost difference. According to the results shown in Table 1, the △costs (Null cost − total cost) of the 10 pharmacophore models generated by the HypoGen algorithm were all larger than 40, indicating that these models are credible. Thus, Hypo_1 should have been selected as the optimal pharmacophore. However, it showed lower predictive ability in the subsequent validation results using the input test set (q^2^ = 0.707). This is in contrast to Hypo_2, which had a more reliable pharmacophore model efficacy (q^2^ = 0.918). Finally, Hypo_2 was selected as the best pharmacophore model, characterized by the lower total cost value (137.012), RMSD (2.37587), correlation coefficient (0.937476), HBA, HYB, HYB, NI, and RA (Figure 3) and the best predictability.

**TABLE 1:** Statistical parameters of the ten pharmacophore hypotheses generated by the HypoGen algorithm.

Hypothesis	R^a^	RMS	Total cost	△cost	Max.Fit	Chemical feature	q^2, b^
1	0.943394	2.26487	133.247	431.397	10.361	HBA, HYD, HYD, and RA	0.707
2	0.937476	2.37587	137.012	427.632	10.2033	HBA, HYB, HYB, NI, and RA	0.958
3	0.921851	2.6455	152.011	412.633	9.2773	HBA, HYD, HYD, NI, and RA	0.867
4	0.915875	2.74076	158.409	406.235	9.59251	HBA, HYD, HYD, and RA	0.763
5	0.88746	3.14624	184.281	380.363	11.4845	HYD, HYD, HYD, NI, and RA	0.694
6	0.88397	3.1919	188.102	367.542	12.2612	HYD, HYD, HYD, NI, and RA	0.717
7	0.872691	3.33312	197.463	367.181	8.74394	HBA, HYD, HYD, HYD, and RA	0.627
8	0.872847	3.33132	197.567	367.077	9.30264	HBA, HYD, NI, and RA	0.884
9	0.870879	3.35523	199.306	365.338	9.28018	HBA, HYD, HYD, and NI	0.770
10	0.866329	3.40959	203.149	361.495	6.97513	HYD, HYD, HYD, and NI	0.738

Null cost = 564.644, Fixed cost = 74.9106, Best records in pass: 6; HBA, Hydrogen bond acceptor; HYB, Hydrophobic; RA, Ring aromatic; NI, Negative ionizable; R^a^: Correlation coefficient of hypothesis based on the training set; q^2, b^: Validation coefficient of the validation result using test ligands.

**FIGURE 4:**
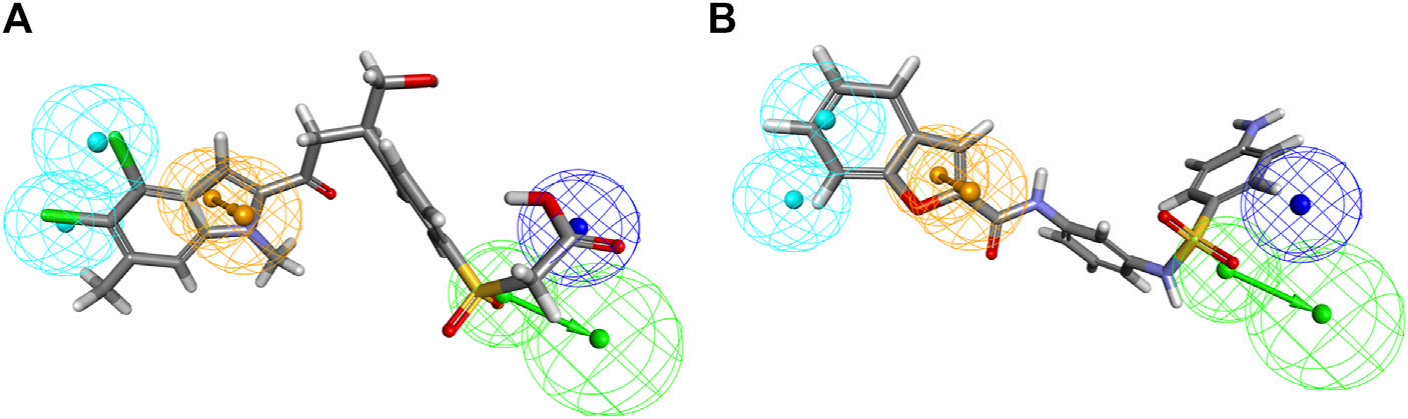
Alignment of the most active and inactive ligands of the training set on the Hypo_2 pharmacophore model. **(A)** compound T1 and **(B)** compound T22.

Inhibitors with known activity were collected from published studies and classified into four groups: the most active (++++, IC_50_ ≤ 0.01 µM); highly active (+++, IC_50_ 0.01–0.1 µM); moderately active (++, IC_50_ 0.1–1 µM); inactive (+, IC_50_ > 1 µM). The experimental and estimated activities of the training set based on Hypo_2 are shown in Table 2. The most active compound in the training set, **T1**, was found to be well-mapped with the essential features of Hypo_2, whereas the inactive compound, **T22**, was not well linked with three chemical features (Figure 4).

**TABLE 2:** Experimental and estimated activity of the training set based on Hypo_2.

Comp. No.	IC_50_ (µM)		Errors	Fit value	Experimental scale	Estimated scale
	Experimental	Estimated				
T1	0.003	0.00135573	−2.21283	8.61813	++++	++++
T2	0.014	0.0159841	1.14172	7.54661	+++	+++
T3	0.015	0.0139266	−1.07708	7.60646	+++	+++
T4	0.028	0.0667427	2.38367	6.9259	+++	+++
T5	0.028	0.0807822	2.88508	6.84298	+++	+++
T6	0.03	0.0152405	−1.96844	7.5673	+++	+++
T7	0.03	0.0826144	2.75381	6.83324	+++	+++
T8	0.051	0.0795133	1.55908	6.84986	+++	+++
T9	0.058	0.0527888	−1.09872	7.02776	+++	+++
T10	0.061	0.138865	2.27648	6.60771	+++	++
T11	0.106	0.124536	1.17487	6.655	++	++
T12	0.123	0.052655	−2.33596	7.02886	++	+++
T13	0.235	0.30066	1.2794	6.27222	++	++
T14	0.25	0.307362	1.22945	6.26265	++	++
T15	0.26	0.805205	3.09694	5.84439	++	++
T16	0.8	0.527419	−1.51682	6.02814	++	++
T17	11.2	0.433289	−25.8488	6.11352	+	++
T18	11.2	13.4728	1.20293	4.62084	+	+
T19	11.7	13.3072	1.13737	4.62621	+	+
T20	11.8	18.7297	1.58726	4.47777	+	+
T21	12.1	13.3406	1.10253	4.62512	+	+
T22	67.7	10.9976	−6.15589	4.709	+	+

### 3.2 Pharmacophore validation

#### 3.2.1 Test set analysis

**FIGURE 5:**
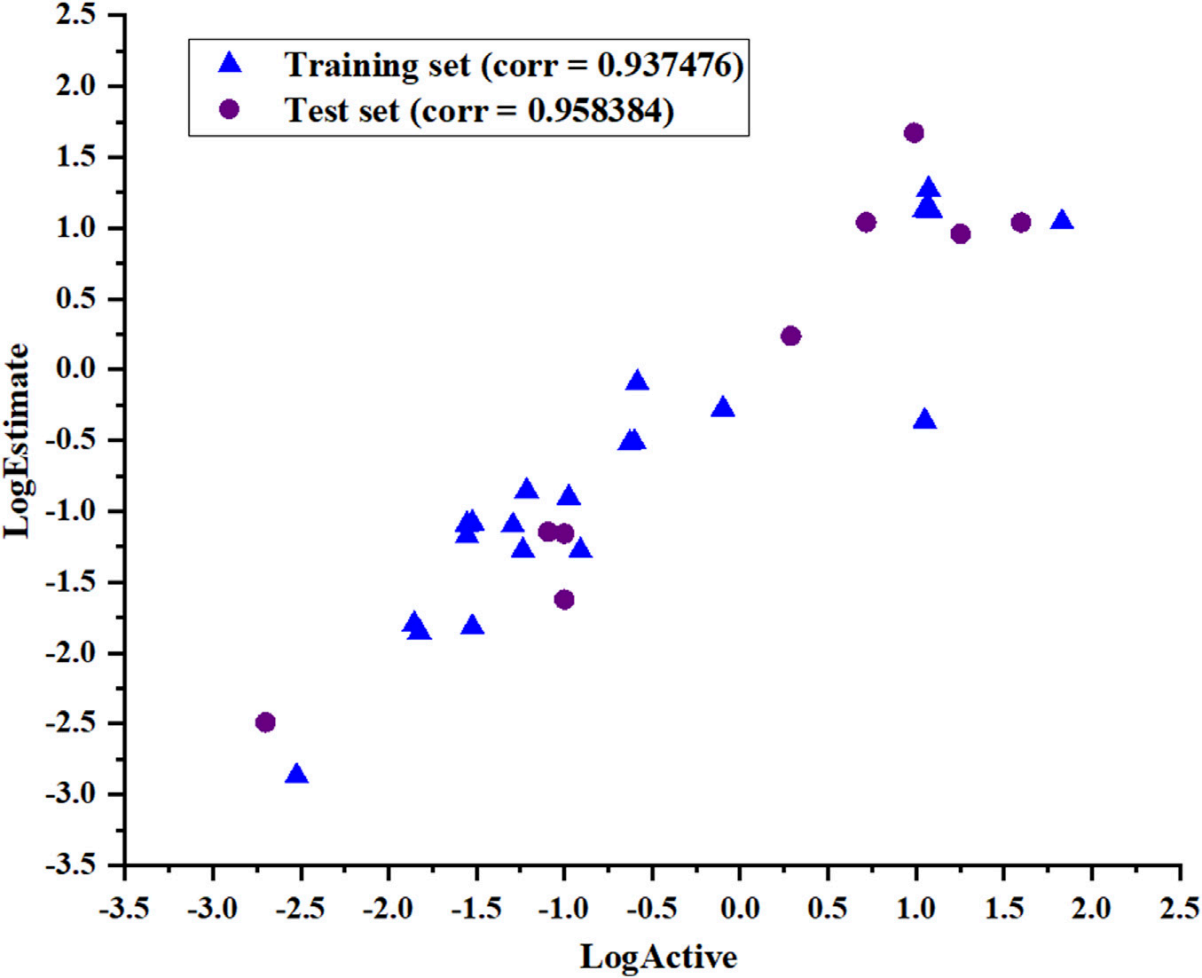
The correlation coefficient (R) of the actual and predicted values of the test set and training set based on the Hypo_2 pharmacophore model.

The ability of Hypo_2 to predict the biological activities of the test set, including nine chemically diverse compounds with IC_50_ values ranging from 0.002 µM to 39.6 µM, was used to assess its dependability. As shown in Table 3, the most active compounds, highly active compounds, and inactive compounds were anticipated to be in the same range as their experimental range, except for two moderately active compounds. Simple regression has been used to analyze the correlation between experimental values and estimates of activity. The results illustrated in Figure 5 show the correlation coefficients between the actual and predicted PHGDH inhibitory activity values for both the training set (R = 0.937476) and the test set (R = 0.958384). With Hypo_2, it was apparent that the test set of nine compounds with known activity had been properly mapped.

**TABLE 3:** Experimental and estimated activity of the test set based on Hypo_2.

Comp. No.	IC_50_ (µM)		Errors	Fit value	Experimental scale	Estimated scale
	Experimental	Estimated				
T23	0.002	0.003228	1.61424	8.2413	++++	++++
T24	0.1	0.023829	−4.19664	7.3732	++	+++
T25	0.1	0.069607	−1.43664	6.90765	++	+++
T26	0.081	0.071526	−1.13246	6.89584	+++	+++
T27	1.95	1.72224	−1.13225	5.51421	+	+
T28	17.9	9.02679	−1.98299	4.79477	+	+
T29	39.6	10.8989	−3.63339	4.71292	+	+
T30	5.2	10.9342	2.10273	4.71151	+	+
T31	9.76	46.8162	4.79674	4.0799	+	+

++++ (IC_50_ ≤ 0.01 µM) represents the most active; +++ (IC_50_ 0.01 µM–0.1 µM) represents high activity; ++ (IC_50_ 0.1 µM–1 µM) represents moderate activity; + (IC_50_ > 1 µM) represents inactive.

#### 3.2.2 Fischer’s randomization test

**FIGURE 6:**
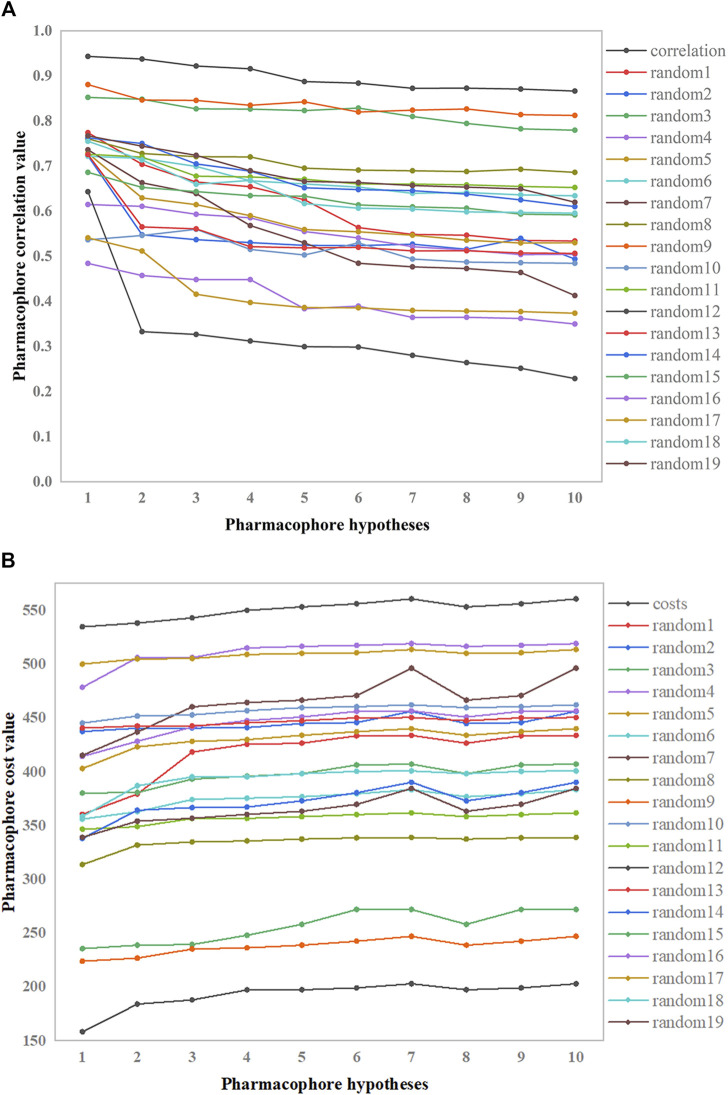
Correlation and total cost values of hypo-PHGDH and 19 random spreadsheets. **(A)** The correlation values; **(B)** The total cost value.

The pharmacophore hypothesis was assessed using the Fischer randomization test (Jacquez and Jacquez, 2002), which was designed with a 95% confidence level. Then, 19 spreadsheets were produced at random. Using this strategy, hypotheses were produced by randomly rearranging the bioactivity values of the compounds in the training set. Figure 6 depicts the differences in correlations (Figure 6A) and cost values (Figure 6B) between the HypoGen and Fischer randomizations. None of the randomly produced pharmacophores performed better statistically than HypoGen.

### 3.3 Virtual screening results and analysis

**FIGURE 7:**
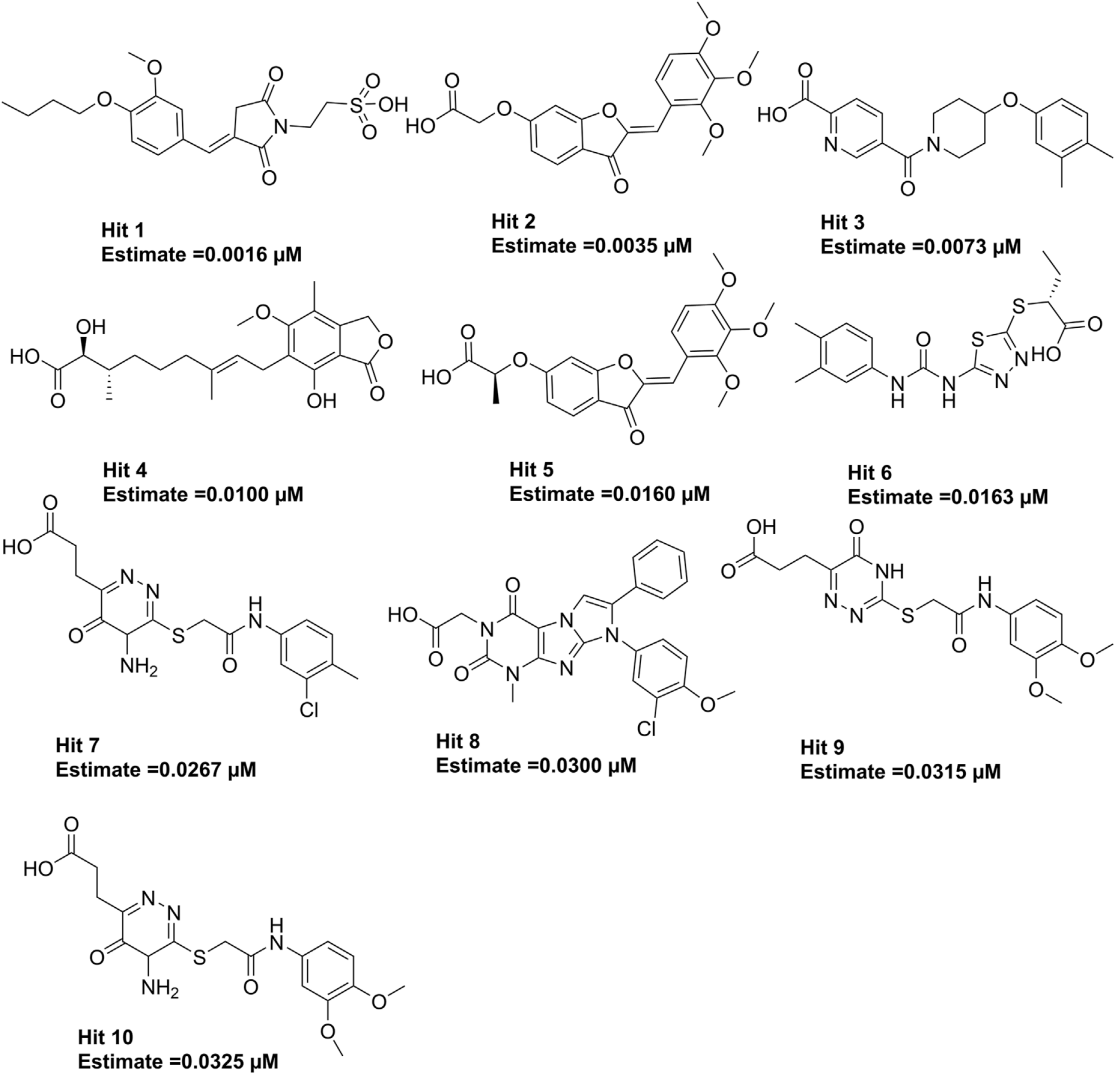
The structures of the top 10 compounds from virtual screening based on Hypo_2.

The 3.04 million compounds from the Lifechemicals database, 460,160 compounds from the Enamine database, and 47,490 compounds from ChemDiv were first filtered by Lipinski and Veber rules (Veber et al., 2002; Lipinski, 2004). The resulting compounds were then virtually screened using the constructed Hypo_2 model, and 1,006 compounds were mapped to Hypo_2. Ten top-ranked compounds with IC_50_ 0.0016 µM–0.0325 µM are shown in Figure 7, and compounds **1**–**4** were selected as hit compounds with estimated IC_50_ values of less than 0.01 µM. The estimated activity and predictive ADME properties of 10 top-ranked compounds are shown in Table 4. All parameters were within Lipinski’s rule of five (ROF) cut-off range for the test compounds resulting from the pre-filter. Toxicity restricts the development of specific compound families at any stage of drug development. Toxicity may take the form of Ames toxicity, carcinogenicity, hepatotoxicity, mutagenicity, or cytotoxicity and is the most noteworthy property of any potential drug candidates. Ideal drug-like compounds would be more highly potent yet not harmful (Ferreira and Andricopulo, 2019). *In silico* prediction of compound cytotoxicity and mutagenicity has drawn great attention from researchers, can assist the early identification of potentially harmful and mutagenic compounds, and can minimize the time and funding involved with hit-to-lead optimization (Kazius et al., 2005; Kramer et al., 2007; Chu et al., 2021). Therefore, we focused on investigating the predictive Ames toxicity, carcinogenicity, hepatotoxicity, cytotoxicity, mutagenicity, and LD_50_ of the top 10 compounds. As shown in Table 5, compounds **1–4** and compounds **7–8** had good ADME properties without any toxicity.

**TABLE 4:** The estimated activity and predictive ADME properties of top 10 screening compounds.

Comp No.	Estimate activity (µM)	MW^a^	NRB^b^	NBD^c^	NBA^d^	ALogP^e^	GI absorption^f^	BBB permeant^g^
Hit1	0.0016	397.44	9	1	7	1.90	High	No
Hit2	0.0035	386.35	7	1	8	2.79	High	No
Hit3	0.0073	354.42	5	1	5	3.08	High	No
Hit4	0.0100	392.44	9	3	7	3.12	High	No
Hit5	0.0160	400.38	7	1	8	3.16	High	No
Hit6	0.0163	366.46	8	3	5	3.75	Low	No
Hit7	0.0267	396.85	8	3	7	1.85	Low	No
Hit8	0.0300	479.87	5	1	6	2.55	High	No
Hit9	0.0315	394.40	10	3	8	0.93	Low	No
Hit10	0.0325	408.43	10	3	9	0.90	Low	No

^a^ Molecular weight of the compounds.

^b^ Number of rotatable bonds.

^c^ Number of H-bond donors.

^d^ Number of H-bond acceptors.

^e^ Prediction by the admetSAR webset.

^f^ Gastrointestinal absorption predicted by SwissADME.

^g^ Blood–brain barrier permeation predicted by SwissADME.

**TABLE 5:** Predictive toxicities of the top 10 screening compounds.

ID Number	Ames toxicity	Carcinogens	Hepatotoxicity	Cytotoxicity	Mutagenicity	LD_50_ (mg/kg)	Predicted toxicity class
Hit1	-	-	-	-	-	600	IV
Hit2	-	-	-	-	-	500	IV
Hit3	-	-	-	-	-	500	IV
Hit4	-	-	-	-	-	500	IV

Note: -: inactive; +: active; toxicity classes are categorized based on the LD_50_ value. Category III indicates toxicity if swallowed, with an LD_50_ value between 50 mg/kg and 300 mg/kg. Category IV indicates harm if swallowed, with an LD_50_ value between 300 mg/kg and 2,000 mg/kg. Category V indicates potential harm if swallowed, with an LD_50_ value between 300 mg/kg and 2,000 mg/kg.

**FIGURE 8:**
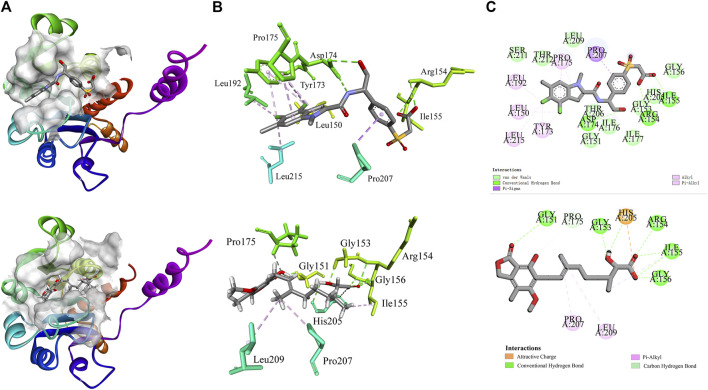
The interactions of co-crystal structure (PHGDH-**T23** complex); docking result diagrams of PHGDH with **hit4** (below). **(A)** Solid ribbon model; **(B)** active sites of amino acid residues represented in the parent color line model; **(C)** 2D interaction diagram.

Molecular docking provides a peripheral vision for investigating ligand and receptor interactions in active pockets. The most active ligand (**T23**) was chosen for active control with a LibDock score of 134.386 kcal/mol and an AutoDock score of −7.1 kcal/mol. Its interactions with PHGDH were first analyzed. As illustrated in Figure 8, the binding affinity of compound **T23** was acquired from two strong hydrogen bonds interacting in the adenine pocket, which was accompanied by a bidentate polar anchor interaction of an -NH group and an -OH group with ASP174. In addition, **T23** was stabilized by two other strong hydrogen bond interactions between residues ARG154 and ILE155. The aromatic ring exhibited Pi–Sigma interactions with PRO207. Using **hit4** as an example for docking analysis, the PHGDH-**hit4** complex was mainly stabilized by six strong hydrogen bond interactions between residues GLY151, GLY153, ARG154, ILE155, GLY156, and HIS205, as well as an attractive charge between HIS205. Moreover, the linker was bound within the hydrophobic cavity consisting of residues PRO207 and LEU192. It was interesting that both **T23** and **hit4** bound into the active pocket of PHGDH in a reversible “V” shape. The docking scores of **hit1** to **hit4** bound to PHGDH were lower than **T23,** especially for **hit1**, which suggested further structure optimization. Meanwhile, derivatives of **hit1** were slightly higher than or comparable to **T23** (Table 6). However, docked scores alone are insufficient to determine the potential of a compound.

**TABLE 6:** Docking scores of **hit 1** to **hit4** and derivatives from **hit1**.

Comp.No.	Fit value	Estimate	LibDock scores (kcal/mol)	AutoDock scores (kcal/mol)
BI-4929 (T23)	8.2413	0.003228	134.386	−7.1
Hit1	8.54524	0.0016	84.4339	−6.2
Hit2	8.19871	0.0035	107.808	−6.3
Hit3	8.10425	0.0073	109.652	−6.7
Hit4	8.03687	0.0100	124.224	−6.6
Hit1-1	8.13017	0.0041	137.977	−7.5
Hit1-2	7.84307	0.0080	116.992	−7.0

### 3.4 MD simulation

**FIGURE 9:**
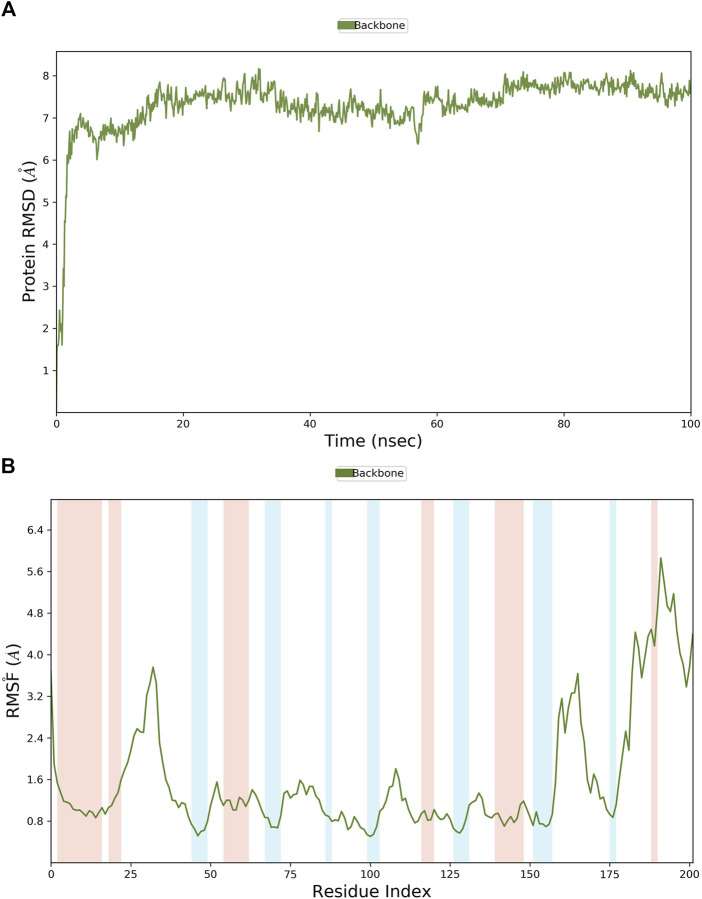
The RMSD and RMSF plots of the empty protein backbone (PDB ID: 6RJ6). **(A)** The RMSD plot of the empty protein; **(B)** The RMSF plot of the empty protein (secondary structure elements: alpha-helical and beta-strand regions are highlighted in red and blue backgrounds, respectively).

MD simulations were employed to further identify the virtual leads. **Hit1** to **hit4** were analyzed and compared with the most potent inhibitor known, **T23**, in terms of the stability and steady nature of the empty protein and the respective contacts and mobility in the pocket of PHGDH. Following the alignment of each protein frame on the reference frame backbone, the atom selection was used to compute the RMSD, which could give insights into the structural conformation by monitoring RMSD in the simulation. When the simulation ends, its fluctuations are centered around a thermal average structure if it has reached equilibrium. Changes of the order of 1–3 Å are acceptable. On the other hand, larger fluctuations suggest that the protein is changing significantly during the simulation. The 100-ns empty protein simulation reached an equilibration phase with RMSD fluctuations in the range of 6 Å–8 Å (Figure 9A). In the Figure 9B plot, peaks indicate areas of the protein that fluctuate during the simulation. Typically, the tails (N-and C-terminal) fluctuate more than any other part of the protein.

**FIGURE 10:**
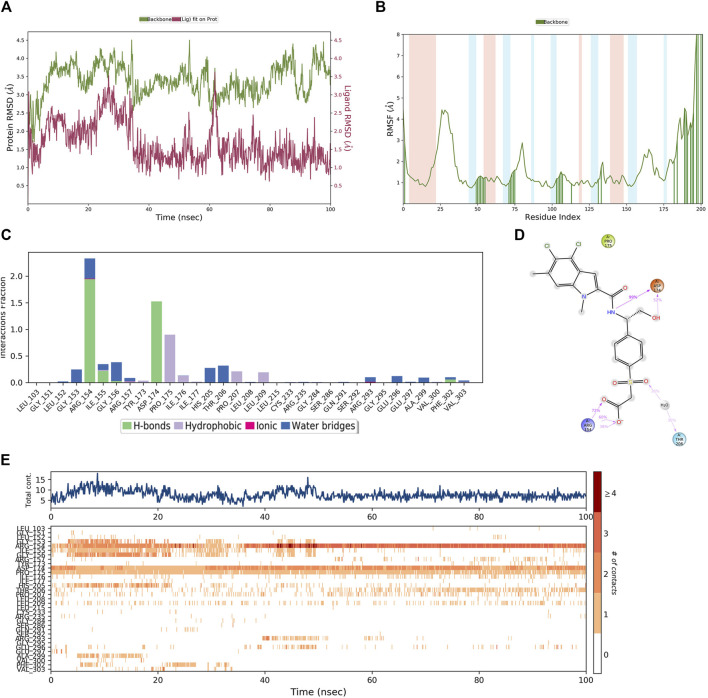
The MD simulation results of the PHGDH-**T23** complex (PDB ID: 6RJ6). **(A)** PHGDH-**T23** complex RMSD plot; **(B)** PHGDH-**T23** complex RMSF plot. **(C)** The main interactions of **T23** with active residues during MD simulation. **(D)** A schematic of detailed **T23** interactions with the protein residues. **(E)** A timeline representation of the PHGDH-**T23** complex contacts.

In order to demonstrate that the screened novel hit compounds have the potential to be more effective inhibitors of PHGDH than the reported available inhibitors, a comparative MD simulation study was conducted using hit compounds and the most potent known PHGDH inhibitor (**T23)** as reported by Weinstabl et al. (2019). The PHGDH-**T23** complex showed initial light fluctuation in RMSD-P within the first 40 ns, which stabilized in the range of 2.5 Å–4.5 Å (Figure 10A). Except for the N-and C-terminal, the P-RMSF fluctuated less than 3 Å. Comparing Figures 9B, 10B shows that protein chain binding to the respective ligand makes the loop region more stable. As shown in Figure 10C, the PHGDH-**T23** interactions are categorized into three types: hydrogen interactions, hydrophobic interactions, and water bridges. During the MD simulation, the residues ASP174 (99%) and ARG154 (72%) of PHGDH exhibited long-time H-bond interaction with the inhibitor **T23**. Water bridging and hydrophobic interactions can also be observed (Figure 10D). The top panel of Figure 10E shows that an average of 10 residues formed contacts with **T23** over the course of the trajectory. Some residues make more than one specific contact with the ligand, which is represented by a darker shade of orange. ARG154, ASP174, and PRO175 are shown with dark orange shades (Figure 10E).

**FIGURE 11:**
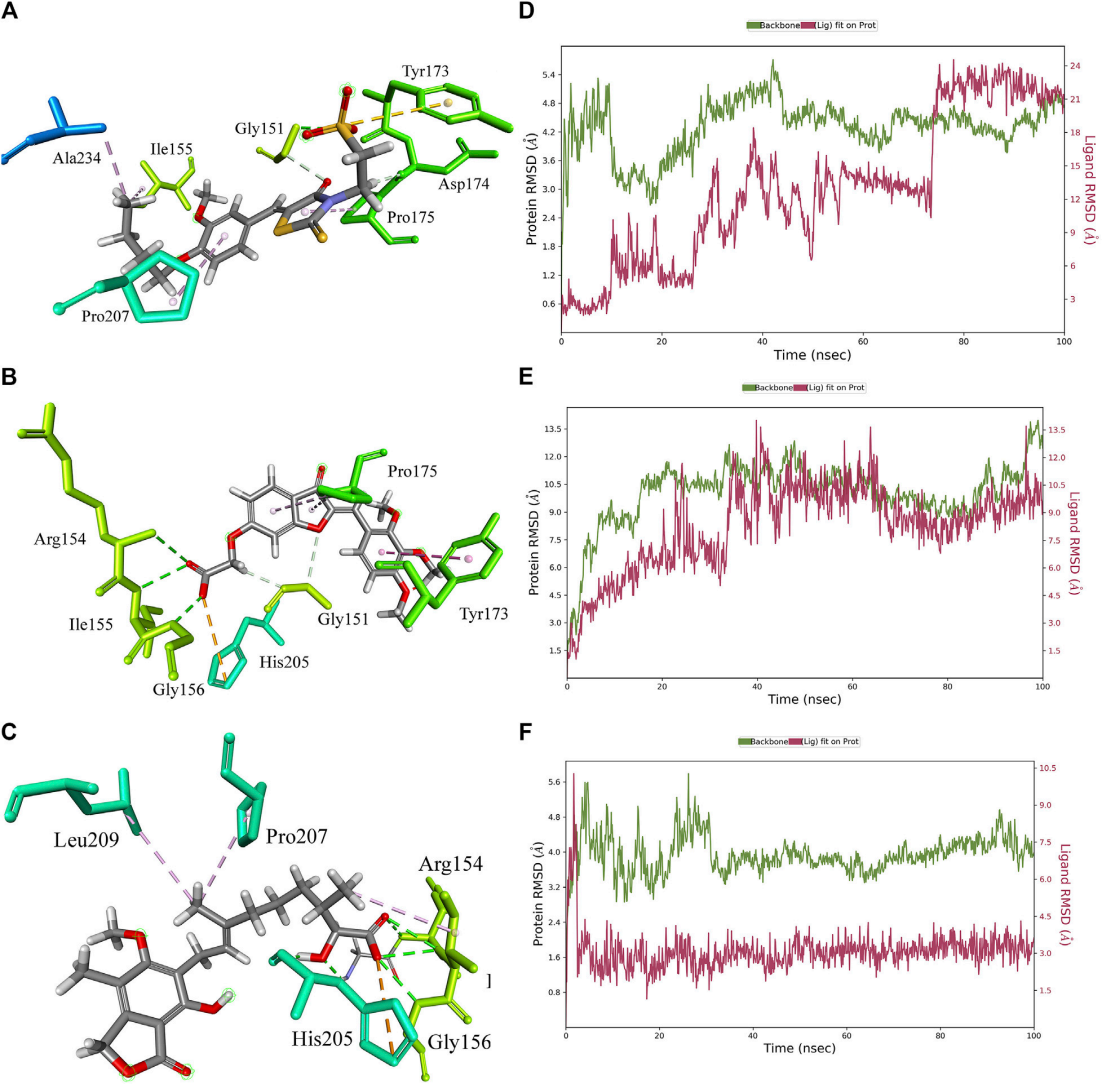
The 3D docking pose and MD simulation results of PHGDH–hit1, PHGDH–hit2, and PHGDH–hit3 complexes. **(A–C)** The 3D docking pose of the hit1, hit2 and hit3 with PHGDH, respectively; **(D–F)** The protein RMSD of PHGDH–hit1, PHGDH–hit2, and PHGDH–hit3 complexes, respectively.

The 3D docking poses and MD simulation of the PHGDH-**hit1**, PHGDH-**hit2**, and PHGDH-**hit3** complexes are shown in Figure 11. The protein backbone RMSD and ligand fit protein initially revealed a large deviation that suggests irregular interactions during the simulations. Throughout simulations, the RMSD fluctuations of ligand fit proteins did not equilibrate or converge, which indicated their weak ligand–protein complex binding behavior and suggests the need for further structural optimization.

**FIGURE 12:**
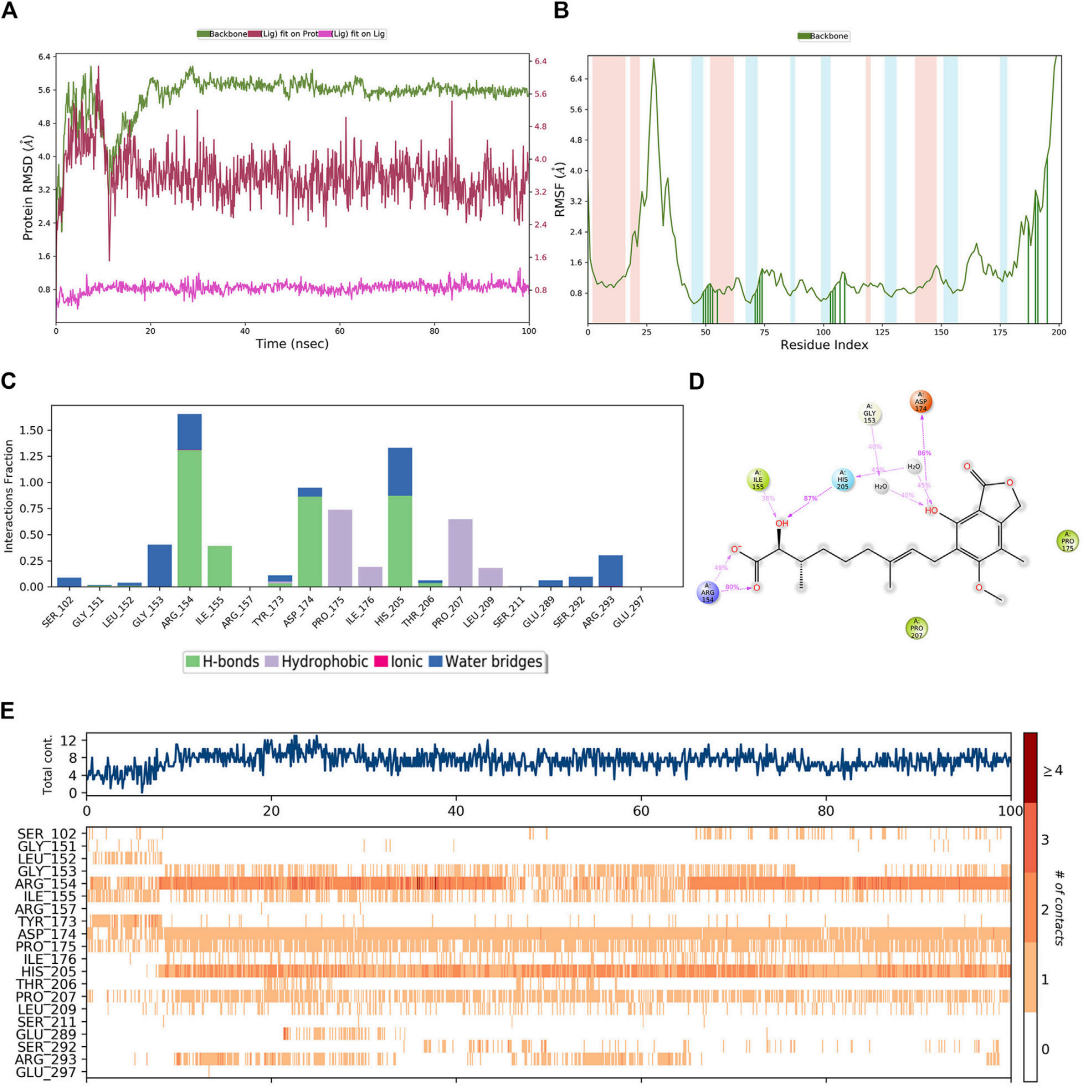
The MD simulation results of the PHGDH–**hit4** complex. **(A)** RMSD plot of the PHGDH–**hit4** complex; **(B)** RMSF plot of the PHGDH–**hit4** complex; **(C)** The main interactions of **hit4** with active residues during MD simulation; **(D)** A schematic of detailed **hit4** interactions with the protein residues; **(E)** A timeline representation of the PHGDH–**hit4** complex contacts.

A similar MD simulation was carried out for **hit4**. The RMSD of the PHGDH-**hit4** complex was steadily maintained from 10 ns to 100 ns, which was approximately 2.4 Å to 4.8 Å (Figure 12A). The residues on the main protein chain showed low fluctuations with an RMSF value of less than 2.4 Å (Figure 12B). The bar histogram displays the four hydrogen bonds between hit4 and residues ARG154, ILE155, ASP174, and HIS205, as well as hydrophobic contacts with residues PRO175, ILE176, PRO207, and LEU209 (Figure 12C). The residues, including ARG154, HIS105, and TASP174, had a major contribution of hydrogen bond interaction to the hydroxyl and carbonyl groups of **hit4** with the percentage of simulation time of 80%, 87%, and 86%, respectively (Figure 12D). Averages of eight residue interactions were maintained until the end of the simulation, as illustrated in the protein–ligand contact plot. Like the PHGDH-**T23** complex, the residues ARG154, ASP174, and HIS205 showed more than one specific contact in 100-ns MD simulations (Figure 12E). **Hit4** might, therefore, be identified as a candidate PHGDH inhibitor for combating various cancers.

### 3.5 Newly designed compounds

**FIGURE 13:**
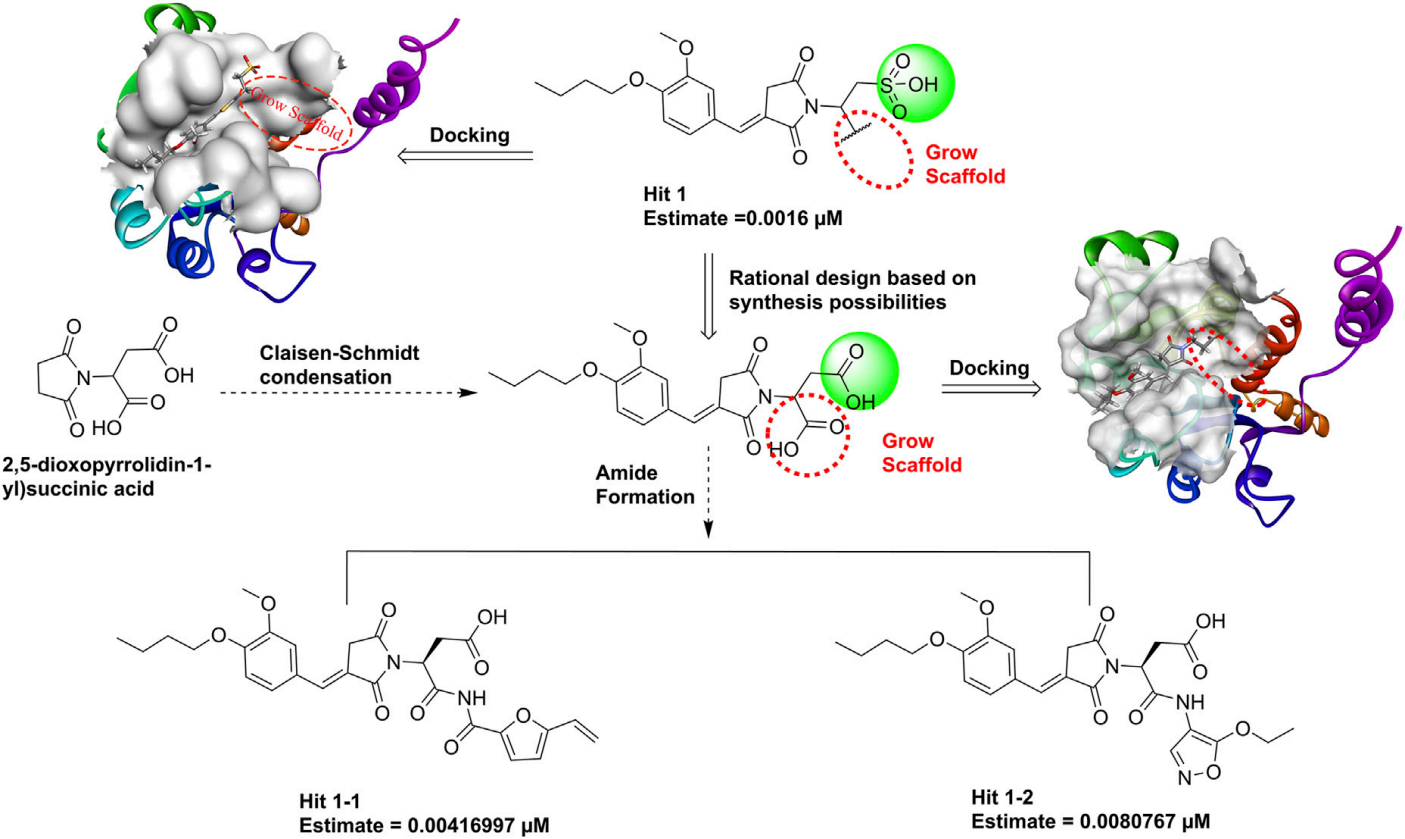
The structures of eliciting hits from **hit1** using Grow Scaffold.

**Hit1**, with the lowest estimated activity value, was selected as the lead compound for subsequent structure optimization. The newly designed derivatives were based on the inclusion of 3D-QSAR model insights and molecular docking. A total of 198 ligands were produced and were subsequently mapped to Hypo_02, and 133 compounds were mapped to Hypo_02. The criterion of estimating activity values less than 0.01 µM was used to further narrow down the scope of virtual hits. Two potential hits (Figure 13) were selected from output ligands. In the next MD simulation, two resulting compounds were evaluated individually.

**FIGURE 14:**
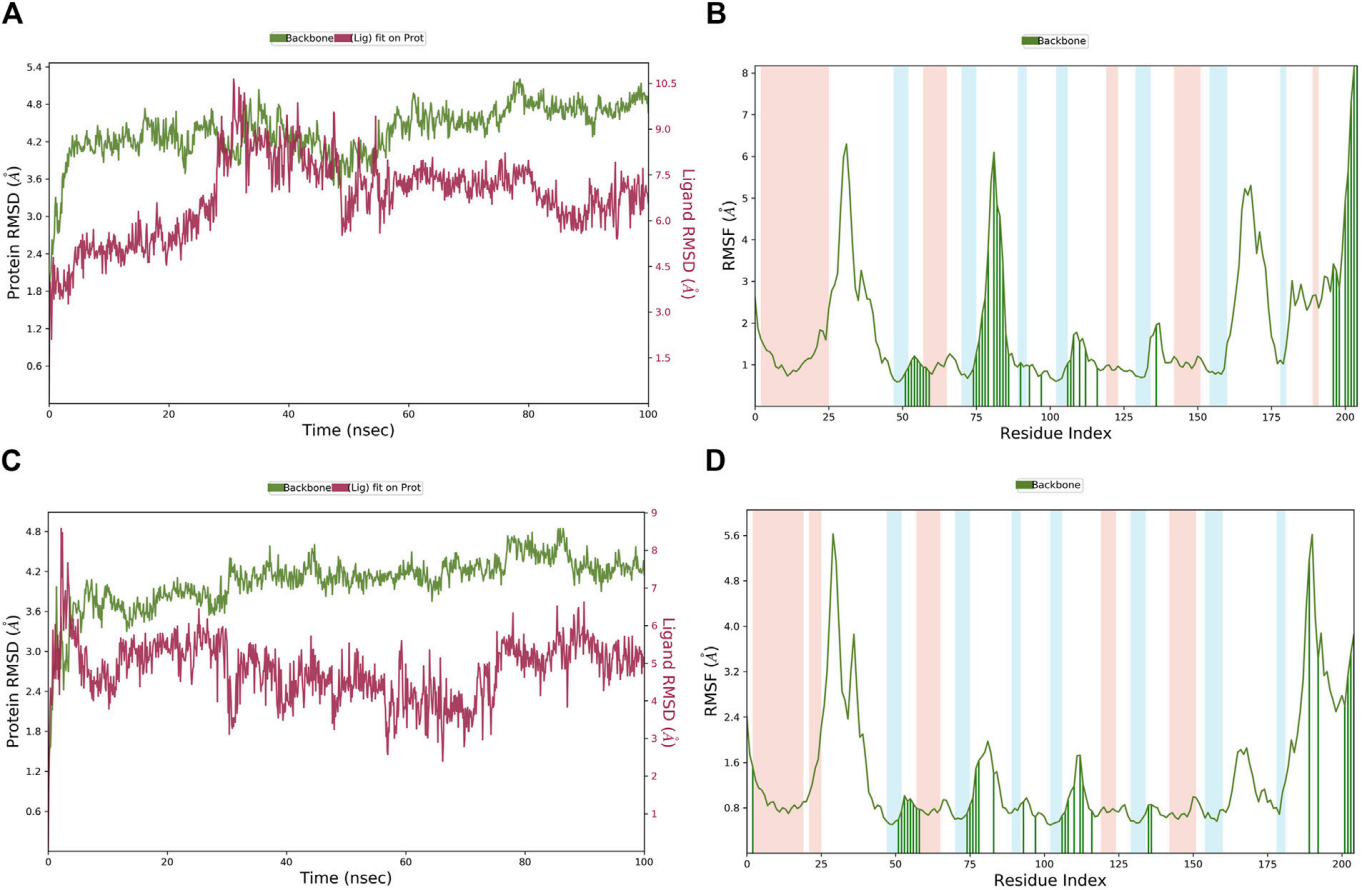
**(A, B)** The RMSD of the PHGDH–**hit1-1** complex and RMSF of the protein plot during a 100-ns simulation; **(C, D)** The RMSD of the PHGDH–**hit1-2** complex and the RMSF of the protein plot during a 100-ns simulation.

The MD simulation results of the PHGDH-**hit1-1** and PHGDH-hit**1**-**2** complexes are shown in Figure 14. Through MD simulation, the RMSD of the protein backbone, Lig_fit_on_Prot, was stabilized in the range of 3.6 Å–5.2 Å, 1.8 Å–4.8 Å, 2.4–4.8 Å and 1.6 Å–4.0 Å, respectively, which indicated the existence of a stable binding pose between protein and ligands. The RMSF of the protein backbone showed high fluctuation at the tails (N- and C-terminals) and loop regions of the protein, especially **hit1-1**. In addition, the other RMSF values interacting with ligands were less than 2.0 Å. The newly designed **hit1**-**1** and **hit1**-**2** showed relatively stable binding poses with PHGDH and good results from the MD simulation studies compared to **hit1**. Therefore, these two newly designed hits could act as potential candidate inhibitors of PHGDH.

## 4 Conclusion

Since PHGDH became an attractive target for cancer research in the past decade, the development of PHGDH-targeted drug discovery has been limited due to the scarcity of reported inhibitors. In this study, a 3D-QSAR pharmacophore with credible predictive was established, which was validated by test set, cost analysis, and Fischer randomization tests. A 3D-QSAR-based high-throughput virtual screening strategy was comprehensively integrated to identify structurally novel PHGDH inhibitors from a commercial database, including approximately 3.54 million small molecules. The drug-like properties of the top 10 compounds were predicted through an *in-silico* study. Safety was considered the primary criterion for manual screening. **Hit1,** with the highest estimated active value, distinguished from the published compounds, was selected for lead optimization, even though unsatisfactory MD simulation results were found. Two hits with an estimated activity of less than 0.01 µM were obtained by lead optimization. Further MD simulation was employed to determine the dynamic binding behavior and binding stability of protein–ligand complexes. The related structural data of identified **hit1** to **hit4** and derivates from **hit1** could be helpful in various cancer-related drug discovery projects.

## Data Availability

The original contributions presented in the study are included in the article/Supplementary Material; further inquiries can be directed to the corresponding authors.
