# Supplementary material for: Identification of novel PHGDH inhibitors based on computational investigation: an all-in-one combination strategy to develop potential anti-cancer candidates
Source: Front Pharmacol. 2024 Aug 27;15:1405350. doi: 10.3389/fphar.2024.1405350 (PMC11383787; doi:10.3389/fphar.2024.1405350)
Supplement: Supplementary file 1 [file DataSheet1.DOCX]

**Identification of novel PHGDH inhibitors based on computational investigation: an all-in-one combination strategy to develop potential anti-cancer candidates**

Yujing Xu ^a,^ ^1^, Zhe Yang ^b, 1^, Jinrong Yang ^a^, Chunchun Gan ^c^, Nan Qin ^a *^, Xiaopeng Wei ^a *^.

1. *Tianjin Key Laboratory on Technologies Enabling Development of Clinical Therapeutics and Diagnostics, School of Pharmacy, Tianjin Medical University, Tianjin, P. R. China*
2. *Tianjin Mental Health Center, Department of Pharmacy, Tianjin Anding Hospital, Tianjin 300222, P. R. China.*
3. *School of Medicine, Quzhou College of Technology, Quzhou 324000, P. R. China.*

^1^ These authors contribute equally to this work.

^*^ Corresponding author at: School of Pharmacy, Tianjin Medical University, Tianjin 300070, China. Email address: [weixiaopeng@tmu.edu.cn](mailto:weixiaopeng@tmu.edu.cn).

**Fig. S1.** The chemical structures of the training set.

**Fig. S2.** The chemical structures of the test set.
